# Effect of an E-learning resource on endoscopists’ proximal serrated polyp detection rate: a randomized controlled trial

**DOI:** 10.1055/a-2240-7823

**Published:** 2024-02-21

**Authors:** David E. F. W. M. van Toledo, Joep E. G. IJspeert, Arne G. C. Bleijenberg, Anne Depla, Nahid S. M. Montazeri, Evelien Dekker

**Affiliations:** 1 Department of Gastroenterology and Hepatology, Amsterdam University Medical Centres, University of Amsterdam, Amsterdam, Netherlands; 2 571165 Amsterdam Gastroenterology Endocrinology Metabolism, Amsterdam, Netherlands; 3 Cancer Centre Amsterdam, Amsterdam University Medical Centres, location Academic Medical Centre, Amsterdam, Netherlands; 4 Department of Gastroenterology, Netherlands Cancer Institute-Antoni Van Leeuwenhoek, Amsterdam, Netherlands; 5 Department of Gastroenterology, DC Klinieken, Amsterdam, Netherlands; 6 Biostatistics Unit, Department of Gastroenterology and Hepatology, Amsterdam University Medical Center, location Academic Medical Center, University of Amsterdam, Amsterdam, Netherlands

## Abstract

**Background** Recent studies demonstrated that a higher proximal serrated polyp detection rate (PSPDR) among endoscopists is associated with a lower risk of post-colonoscopy colorectal cancer (PCCRC) incidence and death for their patients. Our objective was to evaluate the effect of an e-learning resource on PSPDR.

**Methods** We performed a multicenter randomized controlled trial within the Dutch fecal immunochemical test-based colorectal cancer screening program. Endoscopists were randomized using block randomization per center to either receive a 60-minute e-learning resource on serrated polyp detection or not. PSPDR was calculated based on all colonoscopies performed during a 27-month pre-intervention and a 17-month post-intervention period. The primary end point was difference in PSPDR between intervention and control arms (intention to treat) using mixed effect logistic regression modeling, with time (pre-intervention/post-intervention) and interaction between time and arm (intervention/control) as fixed effects, and endoscopists as random effects.

**Results** 116 endoscopists (57 intervention, 59 controls) were included, and performed 27494 and 33888 colonoscopies, respectively. Median PSPDR pre-intervention was 13.6% (95%CI 13.0–14.1) in the intervention arm and 13.8% (95%CI 13.3–14.3) in controls. Post-intervention PSPDR was significantly higher over time in the intervention arm than in controls (17.1% vs. 15.4%, P=0.01).

**Conclusion** In an era of increased awareness and increasing PSPDRs, endoscopists who undertook a one-time e-learning course significantly accelerated the increase in PSPDR compared with endoscopists who did not undertake the e-learning. Widespread implementation might reduce PCCRC incidence.

## Abstract (de)

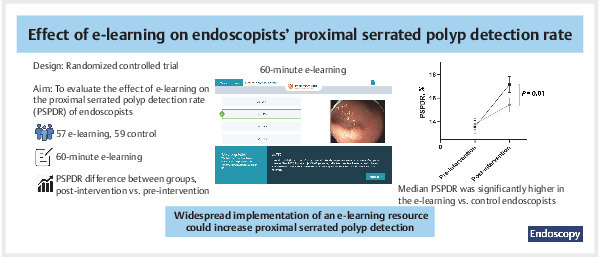

## Introduction

In contrast to the longstanding evidence of adenomas being precursors of colorectal cancer (CRC), evidence for the malignant potential of serrated polyps has only existed for two decades 1 . This major dogma shift has led to improved knowledge about serrated polyps in general 2 ; however, large differences in the detection of serrated polyps suggests that there remains a lack of awareness among many endoscopists 3 4 .

During colonoscopy, detection and removal of all CRC precursors is essential to decrease the risk of CRC. Missed lesions can develop into post-colonoscopy colorectal cancer (PCCRC) 5 . Indeed, most PCCRCs originate from missed or incompletely resected lesions 6 7 . PCCRCs are often right-sided and hypermethylated, features that are consistent with an origin from serrated polyps 8 9 . This is not surprising for at least three reasons. First, serrated polyps are easily missed due to their flat shape and inconspicuous color. Second, serrated polyps are more often incompletely removed than adenomas, owing to their indistinctive borders 10 . Finally, there is still a great deal of misunderstanding about the terminology, classification, and neoplastic risk of serrated polyps 11 .

Hence, the proximal serrated polyp detection rate (PSPDR), defined as the proportion of all colonoscopies in which at least one proximal serrated polyp is detected, has been suggested as a colonoscopy quality indicator and can be used to evaluate the performance of endoscopists in the detection of all clinically relevant serrated polyps. This parameter is preferred over the serrated polyp detection rate as irrelevant distal hyperplastic polyps are now not taken into account, and it is also preferred over the sessile serrated lesion detection rate because the PSPDR is not biased by the interobserver variation among pathologists in the diagnosis of sessile serrated lesions 12 13 . Recently, the additional value of the PSPDR as a quality indicator for colonoscopy has been validated by two studies demonstrating that patients undergoing colonoscopy by an endoscopists with a higher PSPDR have a lower risk for PCCRC and CRC-related mortality 14 15 . A 1 percentage point increase in endoscopists’ PSPDR already lowers the patients’ PCCRC hazard by 7 percentage points and mortality by 3 percentage points 14 15 . Both studies demonstrated the value of the PSPDR alongside the well-known adenoma detection rate (ADR), as individuals treated by endoscopists with both high ADR and high PSPDR had the lowest risk of PCCRC incidence and death.

Thus, PSPDR is a very attractive target for improving colonoscopy efficacy by reducing PCCRC incidence. In a previous study, the PSPDR of 17 endoscopists significantly improved after a simple classroom presentation, but this study was not performed in a randomized setting 16 . Besides, widespread implementation of such a live educational intervention requires resources and experienced teachers. Therefore, easy-to-implement educational interventions, such as an e-learning resource, are warranted. Several experts recently stated the need for evidence that serrated polyp detection rates can be improved within screening programs by use of reasonable interventions 17 . Our objective was to evaluate the effect of an e-learning resource on the PSPDR of endoscopists in a randomized controlled setting.

## Methods

### Study design

This was a multicenter randomized controlled trial performed within the Dutch fecal immunochemical test-based CRC screening program. The research protocol was approved by both the population screening research committee of the governmental National Institute for Public Health and the Environment (RIVM), as well as by our local medical ethical review committee. No participant monitoring was deemed necessary. The study is reported according to the Consolidated Standards of Reporting Trials (CONSORT; see **Table 1s** in the online-only Supplementary material) 18 .

### Participants

Endoscopists were eligible for inclusion if they were accredited within the Dutch CRC screening program. This means that all included endoscopists were subject to strict quality monitoring and auditing throughout the study duration, as described in detail previously 19 . Briefly, each endoscopist has to perform ≥200 colonoscopies per year (screening/symptoms/surveillance), ≥50 polypectomies per year, and achieve a cecal intubation rate of ≥95%, withdrawal time of ≥6 minutes in ≥90% of colonoscopies, adenoma detection rate of ≥30%, removal of ≥90% of all detected polyps, and retrieval for pathologic examination of ≥90% of resected polyps in screening colonoscopies.

All centers participating in the national CRC screening program, excluding nine centers that participated in our previous education study 16 , were invited by emailing one representative endoscopist. Not all email addresses could be requested from the screening organization owing to privacy issues. After randomization, the intervention group and control group were granted access to a digital platform where they had to sign informed consent and complete a short questionnaire about personal characteristics. The digital platform also included the randomization group the endoscopist was assigned to, but only the intervention group could access the e-learning resource. Endoscopists could be excluded for two reasons. First, as we could not retrieve data if no informed consent was signed, endoscopists who did not sign informed consent were excluded from further analysis. Second, as a minimum of 100 colonoscopies were needed to calculate reliable PSPDRs, endoscopists who performed fewer colonoscopies in any period, pre- or post-intervention, were excluded.

Colonoscopy and pathology data were routinely collected as part of standard care in our CRC screening program, as described previously 20 . Colonoscopies were excluded if any of the following applied: 1) a (lesion suspicious for) CRC was detected; 2) cecal intubation was not achieved; 3) Boston Bowel Preparation Scale score was below 6; 4) examination was incomplete and/or prematurely aborted. We linked the colonoscopy data to histopathologic data from PALGA, a database with nationwide coverage of histopathology results. All identifying variables (for patients and endoscopists) were removed by employees of RIVM before the data were sent to our group, in order to comply with privacy legislation of the European General Data Protection Regulation act. Endoscopist characteristics were retrieved from a questionnaire that they completed digitally.

The e-learning resource could be completed between 11 January 2021 and 11 April 2021. Pre-intervention PSPDR was based on all colonoscopies performed by all included endoscopists from 1 August 2018 until 31 December 2020. Post-intervention PSPDR was based on all colonoscopies from 1 April 2021 until 31 August 2022. Lower colonoscopy volumes during the COVID-19 pandemic forced us to extend the collection periods to prevent high dropout rates, because endoscopists did not reach the targeted minimum of 100 colonoscopies within the initially envisioned 12-month period.

Besides signing informed consent, completing the questionnaire, and undertaking the e-learning, no further effort beyond standard care was required from endoscopists, and no contact from the study team occurred during the post-intervention period. As such, participating endoscopists were not reminded of their study participation.

### Randomization

Each representative per center delivered a list of eligible endoscopists who were then block-randomized per center using blocks of 4, 6, and 8 21 . Randomization per center should prevent knowledge from the e-learning resource being transferred from intervention group to control group within the same center. Randomization was performed by an independent person until both arms were equally divided, with an error margin of four endoscopists.

### Procedures

**Fig. 1:**
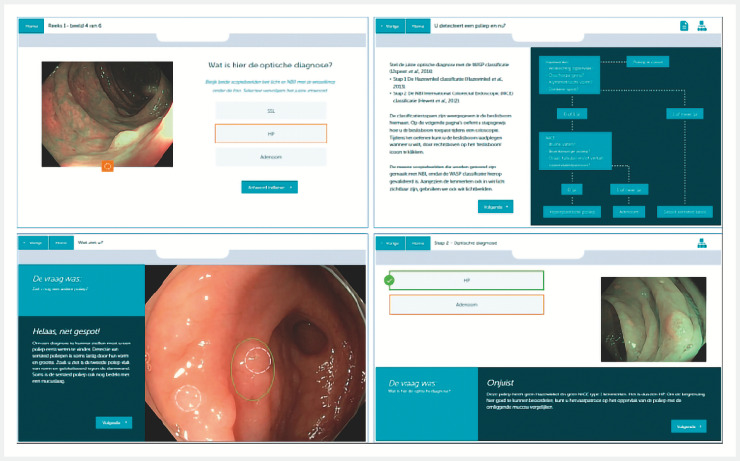
Examples of e-learning slides. Upper left slide: multiple choice question for optical diagnosis, showing polyp with narrow-band imaging; a white-light image of the polyp was also available. Upper right slide: explanation of how to use the flow chart of WASP criteria for optical diagnosis. Lower left slide: participant encircled the incorrect polyp location and received feedback. Lower right slide: training optical diagnosis with feedback on features of the WASP criteria. WASP, classification of the Workgroup on serrAted polypS and Polyposis.

We developed the e-learning resource by collaborating with a commercial third party (Bright Alley, Utrecht, the Netherlands) ( Fig. 1 ). The content was largely based on our previous classroom education module 16 .

The current e-learning resource had the following outline:

1. optical diagnosis exam of 20 polyps by endoscopic images (pre-e-learning exam), with three options: conventional adenoma, sessile serrated lesion, or hyperplastic polyp.
2. 92 pages with interactive educational content, with chapters on why serrated polyps are relevant, detection of serrated polyps, subtypes of serrated polyps, and practicing optical diagnosis of serrated polyps.
3. Estimated time to complete the e-learning was 60 minutes.
4. 19 learning objectives, such as recall the proportion of all CRCs that are derived from serrated polyps, name the three serrated polyp subtypes, appoint Workgroup on serrAted polypS and Polyposis (WASP) criteria to an endoscopic image of a polyp, appoint Narrow-band imaging International Colorectal Endoscopic (NICE) criteria to an endoscopic image 22 , etc.
5. optical diagnosis exam of 20 polyps by endoscopic images (post-e-learning exam). The images and possible answers were identical to those in the pre-e-learning exam, but images were shown in random order.

### Outcomes

The primary end point of the study was the relative difference in PSPDR over time between intervention and control arms. Secondary end points included the relative difference in ADR over time between intervention and control arms. Other secondary end points were the association between PSPDR pre-intervention and absolute difference in PSPDR (diffPSPDR=post-intervention PSPDR minus pre-intervention PSPDR), and the effect of other potential predictors of benefit of e-learning, such as endoscopist’s sex and years of experience. Finally, we compared the median score of the post-e-learning exam with the pre-e-learning exam to test whether endoscopists scored more highly after the e-learning. Primary analyses were done on an intention-to-teach basis, retaining all endoscopists in their initially randomized allocation, and on a per-protocol basis, excluding those endoscopists in the intervention arm who did not complete the e-learning but did complete the informed consent form. Secondary end points were assessed by intention-to-treat analysis.

### Statistical analyses

The sample size calculation was based on our previous study 16 , in which the PSPDR was 12.5% in the intervention arm and 10% in the control arm. Considering 80% power to detect a difference in detection rates of at least 2.5% using a mixed effects model, we calculated with nQuery (version 8.5.1; Statistical Solutions Ltd., Cork, Ireland) that 38 endoscopists performing 100 colonoscopies each were needed per arm. The intracluster correlation was assumed to be 0.005, and alpha was set to 0.05. Considering an endoscopist dropout rate of 20% during the follow-up, 48 endoscopists per arm were needed.

We reported PSPDR and ADR as proportions with 95%CIs. PSPDR was defined as the proportion of all colonoscopies performed in which at least one serrated polyp was detected proximal to the descending colon. ADR was defined as the proportion of all colonoscopies performed in which at least one adenoma was detected.

The primary end point was analyzed with a mixed-effect model including ‘time’ (pre-/post-intervention) and ‘interaction between time and study arm’ (intervention/control) as fixed effects in the model. To capture the heterogeneity between endoscopists, the endoscopists were considered as random effects in the model. We assumed that the random effects have a normal distribution with mean 0 and unknown variance. The same method was used to calculate differences in ADR between the intervention and control arms.

We performed a linear regression analysis to evaluate whether the pre-intervention PSPDR was associated with a benefit from the e-learning resource (diffPSPDR) and to assess the effect of the other potential predictors (endoscopist’s sex and years of experience as a specialist).

To evaluate whether endoscopists scored more highly on the post-e-learning exam vs. the pre-e-learning exam, we used a Wilcoxon signed rank test comparing the median scores.

All analyses were performed using R version 3.6.1 (R Foundation for Statistical Computing, Vienna, Austria). Graphs were created with Graphpad Prism version 9.3.1 (GraphPad Software, La Jolla, California, USA). A *P* value of <0.05 was considered statistically significant.

## Results

### Baseline characteristics

#### Endoscopists

**Fig. 2:**
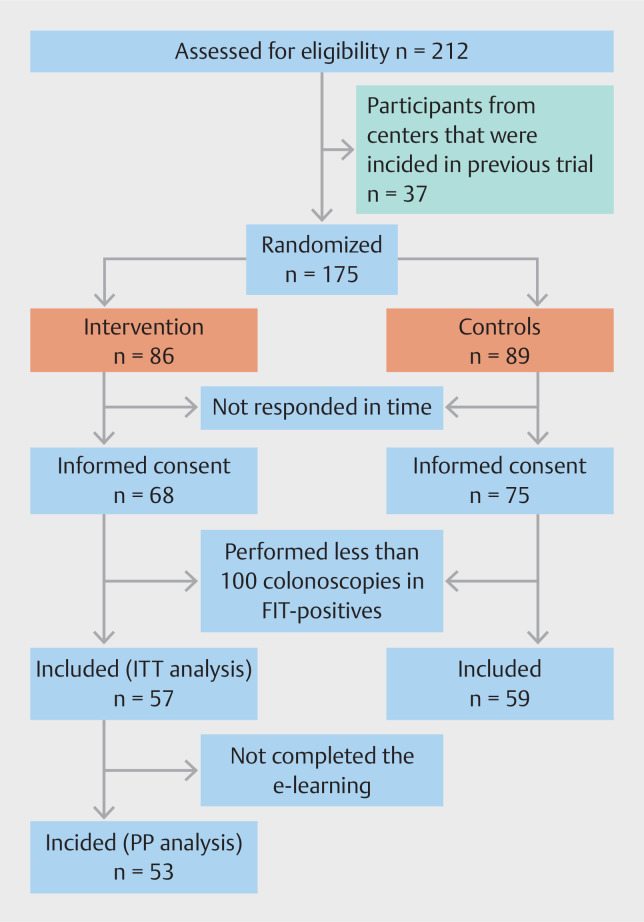
Flow chart inclusions. FIT, fecal immunochemical test; ITT, intention to treat; PP, per protocol.

Of all 175 endoscopists who were screened from 1 September 2020 to 30 November 2020, we randomly assigned 86 to the intervention arm and 89 to the control arm based on the per center randomization ( Fig. 2 ). A total of 68/86 endoscopists (79.1%) in the intervention arm and 75/89 endoscopists (84.3%) in the control arm signed informed consent. At the end of follow-up, we excluded 27 endoscopists who performed fewer than 100 screening colonoscopies pre-intervention or post-intervention. Therefore, 57 endoscopists in the intervention arm and 59 in the control arm were included in the intention-to-treat analysis. For the per-protocol analysis, another four endoscopists were excluded from the intervention group because they did not complete the e-learning.

The median age of all endoscopists (n=116) was 45.0 years (interquartile range [IQR] 40.0–53.0). In total 47 endoscopists (40.5%) were female, 78 (67.2%) worked in a general hospital, 35 (30.2%) in a general hospital and private clinic, and 3 (2.6%) in a private clinic only. During the study period of 45 months, endoscopists performed a median of 514 colonoscopies (range 237–1638). Further details are presented in Table 1 . The optical diagnosis scores in the intervention arm were significantly higher ( *Z=* –3.65, *P<* 0.001, r=0.44) at the post-e-learning exam (median score 6.5 [IQR 6.0–7.0]) compared with the pre-e-learning exam (median score 6.0 [IQR 5.0–6.5]).

**Table:** **Table 1** Characteristics of endoscopists and colonoscopies.

	Overall	Intervention	Control
Included endoscopists, n	116	57	59
Age, median (IQR), years	45 (40–53)	45 (40–49)	46 (40–53)
Sex, female, n (%)	47 (40.5)	25 (43.9)	22 (37.3)
Colonoscopies in study period ^1^ , median (range)	514 (237–1638)	452 (237–1528)	572 (247–1638)
Type of center of endoscopist, n (%)			
General hospital	78 (67.2)	52 (91.2)	26 (44.1)
General hospital + private clinic	35 (30.2)	5 (8.8)	30 (50.8)
Private clinic	3 (2.6)	0	3 (5.1)
Experience, median (IQR), years	11 (5–16)	10 (5–14)	12 (5–17)
Pre-intervention colonoscopies, n	36 076	16 099	19 977
Patient age, median (IQR), years	65 (59–71)	65 (59–71)	65 (59–71)
Patient sex, female, n (%)	15 378 (42.6)	6798 (42.2)	8580 (42.9)
Center, n (%)			
General hospital	28 016 (77.7)	14 684 (91.2)	13 332 (66.7)
Private clinic	8060 (22.3)	1415 (8.8)	6645 (33.3)
Post-intervention colonoscopies, n	25 306	11 395	13 911
Patient age, median (IQR), years	65 (59–70)	65 (59–70)	65 (59–70)
Patient sex, female, n (%)	11 130 (44.0)	4994 (43.8)	6136 (44.1)
Center, n (%)			
General hospital	19 476 (77.0)	9980 (87.6)	9496 (68.3)
Private clinic	5626 (22.2)	1263 (11.1)	4363 (31.4)
IQR, interquartile range. ^1^ Study period was 45 months.			

#### Colonoscopies

In the pre-intervention period, 36 076 colonoscopies were included, of which 28 016 (77.7%) were performed in general hospitals and 8060 (22.3%) in private clinics. The median age of scoped individuals was 65 years (IQR 59–71) and 15 378 (42.6%) were female. Post-intervention, 25 306 colonoscopies were included, of which 19 476 (77.0%) were performed in general hospitals and 5626 (22.2%) in private clinics, with 204 (0.8%) missing data. The median age of scoped individuals was 65 years (IQR 59–70) and 11 130 (44.0%) were female.

### Detection rates

**Fig. 3:**
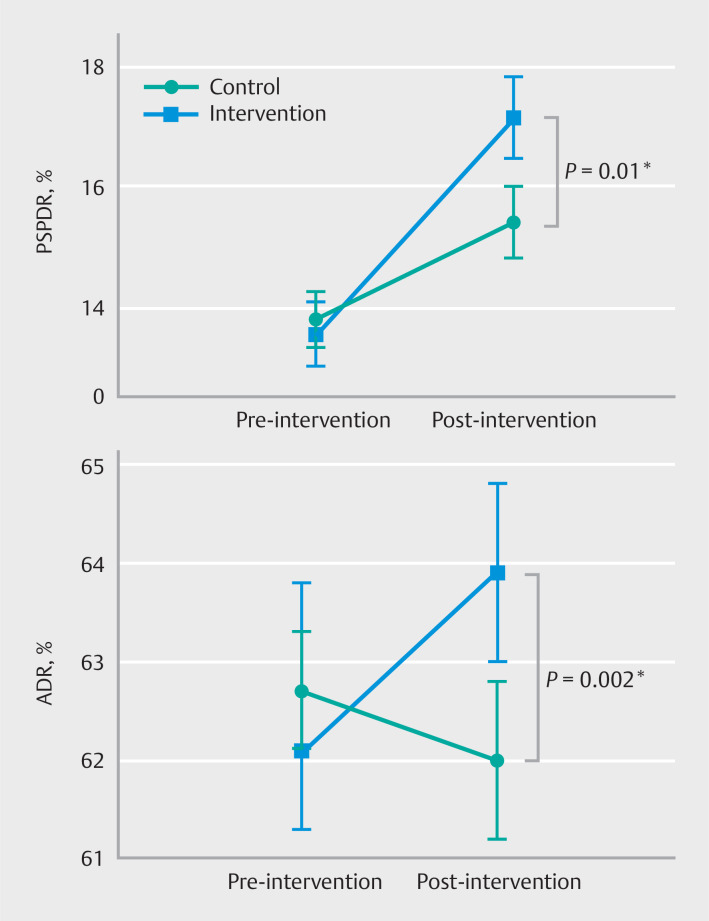
Proximal serrated polyp detection rate (PSPDR) and adenoma detection rate (ADR) pre- and post-intervention. *Statistical comparison using mixed effect logistic regression, with ‘time’ and ‘interaction between time and intervention’ as fixed effects, and ‘endoscopist’ as random effect. Bars represent 95%CIs.

In the intention-to-treat analysis, median PSPDR before intervention was 13.6% (95%CI 13.0–14.1) among endoscopists in the intervention arm and 13.8% (95%CI 13.3–14.3) among endoscopists in the control arm. Median PSPDR after intervention was 17.1% (95%CI 16.5–17.8) in the intervention arm and 15.4% (95%CI 14.8–16.0) in the control arm ( Fig. 3 ). Endoscopists in the control arm had a significant increase in PSPDR over time (odds ratio [OR] 1.12, 95%CI 1.06–1.20, *P<* 0.001) ( Table 2 ). Endoscopists in the intervention arm had a significantly higher PSPDR after intervention compared with the control arm (OR 1.12, 95%CI 1.05–1.19, *P=* 0.01). This was also true in the per-protocol analysis (OR 1.10, 95%CI 1.00–1.20, *P=* 0.04).

**Table:** **Table 2** Mixed effects model for proximal serrated polyp detection rate and adenoma detection rate.

	OR (95%CI)	*P* value
PSPDR		
Time ^1^	1.12 (1.06–1.20)	<0.001
Time : study arm ^2^	1.12 (1.05–1.19)	0.01
ADR		
Time ^1^	0.96 (0.92–1.01)	0.11
Time : study arm ^2^	1.11 (1.04–1.19)	0.002
OR, odds ratio; PSPDR, proximal serrated polyp detection rate; Time, pre-intervention or post-intervention; Study arm, intervention or control; ADR, adenoma detection rate. ^1^ Time’ represents among the control arm the association between pre-intervention and post-intervention. ^2^ Time: study arm’ represents the association between intervention and the control arm post-intervention, controlled for pre-intervention detection rates. Endoscopists were imputed in the model as random effect.		

**Fig. 4:**
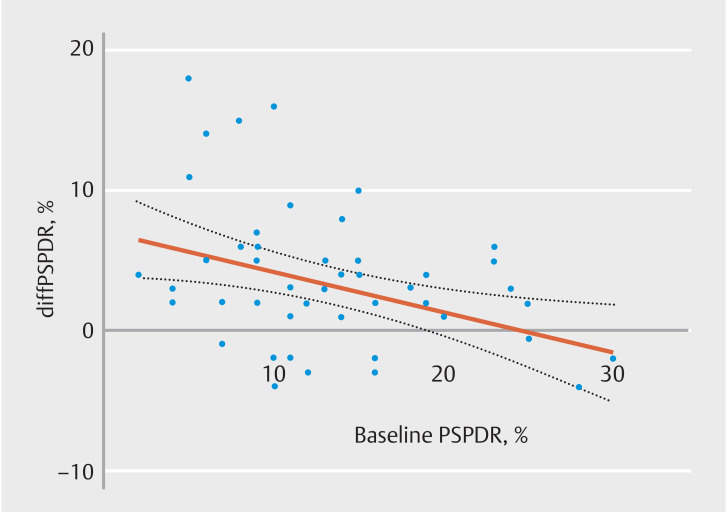
Association between pre-intervention proximal serrated polyp detection rate (PSPDR) and impact of e-learning. diff PSPDR, absolute difference in PSPDR pre- and post-intervention per individual endoscopist.

DiffPSPDR was inversely associated with PSPDR as measured before intervention (β=–0.29, 95%CI 0.62–0.91, *P=* 0.005) ( Fig. 4 ), meaning that a lower PSPDR pre-intervention gave a gradually larger absolute PSPDR rise post-intervention. Endoscopists’ sex and years of experience were not significantly correlated to the diffPSPDR ( Table 3 ).

**Table:** **Table 3** Association between endoscopist characteristics and absolute improvement in proximal serrated polyp detection rate after intervention.

	Linear regression	*P* value
Years of experience	0.001	0.45
Sex	0.003	0.82

The ADR of the intervention arm increased from 61.8% (95%CI 61.0–62.5) pre-intervention to 63.7% (95%CI 62.9–64.6) post-intervention, which was a significant increase over time compared with the control arm (OR 1.11, 95%CI 1.04–1.19, *P=* 0.002) ( Table 2 , Fig. 3 ). ADR in the control arm showed a nonsignificant decrease from 62.7% (95%CI 62.0–63.3) to 62.0% (95%CI 61.2–62.8) (OR 0.96, 95%CI 0.92–1.01, *P=* 0.11).

## Discussion

In this randomized controlled trial, we demonstrated that endoscopists who undertook e-learning of 60 minutes had a significantly higher PSPDR at 17 months of follow-up compared with control endoscopists who did not undertake e-learning. Endoscopists with lower pre-intervention PSPDRs gained more benefit from the e-learning. Bearing in mind the negative association of PSPDR with PCCRC incidence and related mortality, an easily implementable e-learning resource could contribute to lower PCCRC incidence and mortality worldwide.

This was the first randomized controlled trial evaluating the effect of an educational intervention aimed at improving PSPDR. This study was carried out within our organized national screening program. This setting ensured a high quality of prospectively recorded data, and a high quality of endoscopists and pathologists as described previously; these factors are crucial when evaluating polyp detection rates 19 .

Before we can draw definite conclusions on the effectiveness of our e-learning resource to increase endoscopists’ PSPDRs, several issues should be discussed. When studying an effect of education over time, it is challenging to attribute a difference in outcomes to a single effector, such as e-learning in the current study. Two previous papers studied the use of educational interventions in improving endoscopists’ PSPDR; classroom training including 17 endoscopists was shown to be effective 16 , whereas an educational poster in the endoscopy room was not 23 . Neither of these studies was conducted in a randomized controlled setting, which is the most appropriate design to evaluate a potential effect, ensuring equal conditions in two arms. To achieve this aim in the current study, endoscopists were unaware of their role until they accessed the digital platform, so endoscopists in both arms were equally motivated to access the digital platform to sign informed consent and were prepared to undertake the e-learning. Only after signing informed consent were endoscopists made aware of their allocation to the intervention or control arm. We also kept communication with endoscopists uniform. Moreover, because of automated registration of colonoscopy reports, endoscopists were not reminded of their study participation and thus worked according to their usual routine practice. These conditions reduced the possibility that either the trained or the untrained arm were discrepantly motivated to detect proximal serrated polyps, a response also known as the Hawthorne effect 24 . Additionally, we applied randomization per center in order to restrict the sharing of education from trained to untrained endoscopists who worked within the same center.

An important contributing factor to our results was the increasing awareness of serrated polyps among the Dutch gastrointestinal community in general through continuing education. In fact, this increased awareness is reflected by our results demonstrating a significant increase of PSPDR over time in the control arm (OR 1.12, 95%CI 1.06–1.20). This gradual increase was also encountered in the last years of our longitudinal study of the complete screening program (+0.5%/year), as well as in two US studies (+0.4%/year) 14 25 26 . In the initial phase (2014–2017) of our screening program, the PSPDR was more or less stable, at about 10%; thereafter, awareness of proximal serrated polyps seemed to increase 14 16 . As most other countries around the world measure a PSPDR lower than 10% 13 25 26 27 on average, much room for improvement remains, even in a setting where the awareness is already fair.

Another major finding of this study was the linear association of PSPDR pre-intervention with the absolute PSPDR difference before and after the intervention. In other words, endoscopists with the lower PSPDRs pre-intervention benefitted more from the e-learning than endoscopists with higher PSPDRs pre-intervention. Endoscopists’ features such as years of experience or sex were not associated with improvement in PSPDR after e-learning. A subsequent question would be how to define a low or high PSPDR. Looking at the widely varying PSPDRs in other studies, from 2.8% to 18%, defining these performance benchmarks can be rather complicated 3 27 28 29 . PSPDRs have been associated with endoscopist-dependent factors such as specialty of endoscopists, year of completion of specialist training, and procedural volume 30 . In addition, a meta-analysis showed that serrated polyp prevalence varies around the world, with higher prevalence rates in the USA and Europe, and lower rates in Asia 31 . In summary, PSPDR benchmarks may have to be tailored to geographic region or country.

It was not only the PSPDR that increased over time, but also the ADR, which was significantly higher in endoscopists of the intervention arm compared with controls. Although the e-learning was dedicated to serrated polyps, the WASP criteria included in the resource also covers differentiation between adenomas and serrated polyps 22 . To practice this classification, endoscopists in the intervention arm exercised their optical diagnostic skills as part of the e-learning course, reviewing endoscopic images of adenomas, hyperplastic polyps, and sessile serrated lesions. This might have resulted in improved recognition of adenomas and an increase in ADR for endoscopists in the intervention arm. It seems less likely that this higher ADR was caused by the Hawthorne effect as, apart from the e-learning, endoscopists from both arms were treated similarly.

For the interpretation of our results some limitations need to be addressed. In our design, randomization was performed at the level of endoscopy center, while informed consent for study participation and retrieval of data were gathered per individual endoscopist. Owing to privacy issues, data of endoscopists who worked in a randomized center, but did not complete the informed consent form, could not be evaluated. However, allocation to the study arm was only made clear to participants after completion of the informed consent form; therefore, we believe that this randomization approach did not cause significantly biased results. Second, endoscopists who completed the informed consent form, but did not perform at least 100 colonoscopies pre- or post-intervention, were also excluded, both from the intention-to-treat analysis and from the per-protocol analysis. As colonoscopy volume within this period does presumably not correlate with each endoscopist’s motivation to detect proximal serrated polyps, this limitation does not seem to affect our results. Finally, although our finding that lower performers seemed to gain more benefit from the e-learning could also be explained by a statistical phenomenon called regression to the mean, it does not affect our conclusions.

Our e-learning resource was an effective, low-cost, 60-minute educational tool that could be easily implemented in many practices worldwide with the aim of increasing the PSPDR in daily colonoscopy practice. The e-learning was effective in a strict setting of accredited endoscopists who are monitored for quality and have relatively high PSPDRs. This e-learning resource could also be added to the accreditation and auditing process of endoscopists within the screening program. As we demonstrated that endoscopists with a lower PSPDR benefited more from the e-learning, and PSPDRs may be lower in other settings, the impact of our e-learning resource could be greater in these groups and settings. However, to enable PSPDR monitoring, accurate registration of serrated polyps is required, and in most international endoscopy units this is not yet routine. As such, accurate registration of all quality parameters, including PSPDR, is of utmost importance to assure and improve colonoscopy quality.

In conclusion, in this randomized controlled trial, we demonstrated that within a quality-assured setting with growing awareness of serrated polyps, one-time e-learning was effective at increasing the PSPDR. Endoscopists with low PSPDRs had the highest benefit of training. These results support widespread implementation of such an e-learning resource to contribute to a decrease in PCCRC incidence and mortality.

## Supplementary Material

Supplementary Material Zusatzmaterial
